# The Development of Emotion Recognition Skills from Childhood to Adolescence

**DOI:** 10.3390/ejihpe15040056

**Published:** 2025-04-08

**Authors:** Marialucia Cuciniello, Terry Amorese, Carl Vogel, Gennaro Cordasco, Anna Esposito

**Affiliations:** 1 Department of Psychology, Università degli studi della Campania “Luigi Vanvitelli”, CE 81100 Caserta, Italy; terry.amorese@unicampania.it (T.A.); gennaro.cordasco@unicampania.it (G.C.); anna.esposito@unicampania.it (A.E.); 2 Trinity Centre for Computing and Language Studies, Trinity College Dublin, The University of Dublin, D02 Dublin, Ireland; vogel@tcd.ie

**Keywords:** facial emotion recognition, children, pre-adolescents, adolescents, stimuli’s ethnicity

## Abstract

This study investigates how the ability to recognize static facial emotional expressions changes over time, specifically through three developmental stages: childhood, preadolescence, and adolescence. A total of 301 Italian participants were involved and divided into three age groups: children (7–10 years), pre-adolescents (11–13 years), and adolescents (14–19 years). Participants completed an online emotional decoding task using images from the Child Affective Facial Expression (CAFE) database, depicting anger, disgust, fear, happiness, sadness, surprise, and neutrality, conveyed by children of different ethnicities (African American, Caucasian/European American, Latino, and Asian). Results indicated that female participants generally exhibited a higher emotion recognition accuracy than male participants. Among the emotions, happiness, surprise, and anger were the most accurately recognized, while fear was the least recognized. Adolescents demonstrated a better recognition of disgust compared to children, while pre-adolescents more poorly recognized neutrality compared to children and adolescents. Additionally, this study found that female facial expressions of disgust, sadness, and fear were more accurately recognized than male expressions, whereas male expressions of surprise and neutrality were better recognized than female expressions. Regarding the ethnicity of facial expressions, results revealed that ethnicity can be better or more poorly recognized depending on the emotion investigated, therefore presenting very heterogeneous models.

## 1. Introduction

The ability to accurately recognize and interpret emotional facial expressions is crucial for engaging and being adaptively responsive during social interactions. Studies define this ability as emotion recognition (ER), which involves inferring the internal emotional state expressed by others based on external signals. These external signals can be represented, for example, by a pattern of facial muscle movements, body language, gestures, or vocal prosody. The accuracy in interpreting external signals allows an individual to differentiate a specific state from a list of other emotional categories (Ruba & Pollak, 2020a). Castro and colleagues observed that ER is a process that occurs in the presence of specific skills. According to them, an individual should have acquired the ability to be aware that an emotion has been conveyed, label prototypical and atypical emotions, and process contextually relevant information to correctly finalize the emotion identification process (Castro et al., 2016). Beyond the capabilities that underlie ER, it is worth highlighting that ER itself intervenes in other important processes, such as cognitive empathy, which translates into the ability to rationally understand and recognize an emotional state and to adopt another perspective (Bons et al., 2013). Moreover, the development of ER abilities divergent from those considered “typical” are often associated with cases of neglect, institutionalization, or abuse (Moulson et al., 2015; Bick et al., 2017). Conversely, individuals in whom no difficulties or deficits have emerged show a greater mastery in recognizing emotions expressed by others, achieve a better academic performance, have higher self-confidence, and reveal greater social competence (Izard et al., 2001; Goodfellow & Nowicki, 2009; Torres et al., 2015; Bardack & Widen, 2019). Curiously, although the ability to discriminate facial expressions seems to have an early onset (in the first 7 months of life) (Bayet et al., 2014; Palama et al., 2018), the acquisition of recognition skills is progressively refined during childhood and adolescence (Ewing et al., 2017). Therefore, high levels of ER are not fully achieved until late childhood or adolescence (Gao et al., 2010; Rodger et al., 2015). This aspect, however, raises some debate in the scientific field due to conflicting findings. For instance, a study conducted by Griffiths and colleagues revealed that children and adult participants showed a similar accuracy in facial emotion recognition scores, indicating no evidence of an age-related advantage in either group (Griffiths et al., 2015). Despite that this is still an open issue, there is a general consensus on the thesis that each basic emotion follows a specific developmental trajectory. This underscores the importance of considering the ER of facial expressions individually, rather than as a linear and simultaneous process. According to some studies that draw on the constructionist approach, human beings do not possess the innate ability to discriminate between distinct emotional categories, but this ability is acquired through the experience and perception of general fundamental affective states. This gradually leads to a greater accuracy, allowing the discrimination of specific emotional categories based on the acquisition of conceptual knowledge related to emotions (Hoemann et al., 2019; Shablack & Lindquist, 2019). In this regard, Widen’s study is noteworthy; the author investigated the specific developmental trajectories associated with children’s freely inferred labels of facial expressions. The basis is the hypothesis that infants firstly interpret emotional cues by classifying them into valence-based categories (i.e., emotional cues that are considered pleasant or unpleasant). Secondly, as they gradually refine their abilities during infancy, they become able to infer discrete emotion categories. This process has been called the “broad-to-differentiated” pattern (Widen & Russell, 2008; Widen, 2013). A remarkable study by Widen and Russel on 168 children aged 2 to 5 years analyzed the development of basic emotional recognition categories (fear, happiness, sadness, and anger) through three tasks: labeling animals (to assess the preliminary ability to produce labels), the free labeling of facial expressions (to measure the spontaneous ability to infer emotions), and a categorization task (to classify emotions into specific “boxes”). The results show that emotional categories are initially broad, grouping emotions of the same valence (positive or negative). Over time, these categories gradually become more refined. It is interesting to report what happened for the emotional category of “fear”. Before using the label “fear”, children had already started to categorize fear faces and exclude positive faces from the fear category. However, after using the label, they still included other negative faces. Therefore, this pattern of gradual narrowing was observed for all the emotional categories investigated (Widen & Russell, 2008). In a further emotion recognition task, in which children were expected to validate categories of emotional stimuli by associating free labels (i.e., not based on predetermined response options), Widen found that the first emotional facial expressions that children around two years of age consistently identified were happy smiles. As their expressive vocabulary increased, starting at age three, children showed a higher number of emotional terms that allowed them to correctly label frowns and sad cries. It is from around age nine that children correctly decode nose wrinkles attributable to the emotional category of disgust during tasks that involved spontaneous labeling (Widen, 2013), often incorrectly interpreted as angry glares, as also confirmed by the results of a previous study that compared the recognition performances of 22 young adults with those of 84 children (aged 4 to 9 years). This investigation highlighted developmental differences in emotion recognition between children and adults. In the presence of both disgusted and angry expressions, the children involved labeled the expressions in both cases with “anger”. The reasons provided made this study even more interesting as children gave answers for disgusted faces very similar to those given for angry faces. In contrast, most adults labeled the disgusted face accurately and gave responses consistent with the emotion of disgust (Widen & Russell, 2010). As it is evident, there are countless scientific contributions involving children and adults in emotion recognition tasks. Surprisingly, the same interest does not seem to be directed towards pre-adolescents. Investigating pre-adolescents as a distinct group acquires fundamental importance for the current study in the perspective of the transition phase between childhood and adolescence; this is a particular phase too often neglected although it is characterized by significant changes from a cognitive, emotional, physical, and biological point of view, and these changes can often have repercussions on behavior, identity, and social interaction. Understanding the dynamics of pre-adolescents by identifying them as a distinct group also allows us to undertake the development of more effective educational, social, and psychological interventions, specific to their needs and challenges. To our knowledge, in the scientific field, this aspect has not received due attention. The few studies that have investigated it report conflicting results, thus highlighting the need to find answers. In this sense, the study by Lawrence and colleagues is noteworthy; on the one hand, it suggests that the recognition of basic emotions during childhood and adolescence could be influenced by the level of pubertal development (Lawrence et al., 2015), and the study by Motta-Mena & Scherf, on the other hand, states that although the ability to recognize basic emotional expressions improves with age puberty alone does not seem to play a significant role in this improvement (Motta-Mena & Scherf, 2017). Therefore, this contrast underscores the need to differentiate between the effect of age and the potential role of puberty in emotional development.

### 1.1. An Overview of Some of the Factors That May Influence the Development of ER

Over the years, emotion recognition has continued to captivate the scientific community, driving efforts to address unresolved questions surrounding this pivotal topic. To gain a deeper understanding of how these abilities evolve it could be interesting to cite some recent studies carried out during the COVID-19 crisis, which also allows us to investigate the potential environmental impact due to this particular and devastating condition that has inevitably led to restrictions, changes, and isolation and thus determining potential implications in social interactions. Among the most important interventions introduced to counter the spread of COVID-19, the obligation to wear face coverings is noteworthy. This has led researchers to wonder about the potential effects of the influence of the occlusion of parts of the face on the emotional inferences that children make during social exchanges. Ruba and Pollak’ study involved a sample of school-age children (aged 7 to 13). The results of this study highlight that even in conditions in which some parts of the face (such as the mouth and eyes) were covered, children were equally accurate in providing inferences about emotions (Ruba & Pollak, 2020b). Another relevant investigation delves into the effects related to the color of the mask. To conduct this study, the performances of 24 adults and 27 children were compared with respect to different conditions. Findings of this study highlight that wearing a mask does not affect both children’s and adults’ ability to process facial emotional information (Gil & Le Bigot, 2023). To our knowledge, there are many variables that can influence the ER process; ethnicity and gender are undoubtedly among these. Regarding ethnicity, an own-race advantage in recognizing emotions has been found. In a study conducted by Kang and Lau, when European Americans and Asian Americans were asked to validate authentic expressions, a mutual in-group advantage emerged for both groups, despite living in the same country. However, these results should be interpreted with caution, as this effect did not occur in the acted-out expressions condition, suggesting it could be related to the type of stimulus (Kang & Lau, 2013). Contrasting and more recent findings did not report this own-race advantage, emphasizing the need for further investigations on this topic (Lawrence et al., 2015). Several studies, on the other hand, have given priority to exploring sexual differences in facial emotion recognition, revealing a female advantage (Reyes et al., 2018), which remains constant throughout the lifespan and progressively attenuates with increasing age (Olderbak et al., 2019). Going into more detail, evidence regarding sex differences in facial expression recognition reports that these are transient and unstable throughout development (Mancini et al., 2013; Mancini et al., 2018).

### 1.2. Aims of the Present Study

Building on the topics covered in the previous brief review, there is a need for a clearer and more comprehensive identification of the gaps in the literature that this research attempts to address to better define its objectives. Few studies have focused on the ability of pre-adolescents to recognize emotions, and even fewer have examined sex differences across the three age groups under investigation. Regarding the stimuli, the own-race advantage remains largely unexplored, as does the potential influence of the stimulus’ sex on ER. Therefore, the present study aims to observe the development of this process through the involvement of children, pre-adolescents, and adolescents who were required to recognize facial expressions of anger, disgust, fear, happiness, sadness, surprise, and neutrality conveyed by static images of children belonging to different ethnic groups (African American, Caucasian/European American, Latino, and Asian). Regarding the choice to include neutrality in the task, it is useful to share the following considerations. Traditionally, a neutral affect is often conceptualized as a bipolar dimension or two unipolar dimensions with either a positive or negative valence (Russell, 2003; Larsen et al., 2009). An intriguing study by Gasper and colleagues provides a comprehensive examination of the neutral affect, revealing divergent perspectives among researchers: some consider it a baseline state in emotion regulation, while others equate it with low-arousal emotions, such as boredom or relaxation. Based on these insights, the current study aims to highlight the importance of clearly defining the neutral affect by conducting an experiment also dedicated to help determine whether it is a distinct construct or occurs in overlap with other constructs. For the authors, the neutral affect should be understood as the presence of neutrality, rather than simply being reduced to the absence of a positive or negative valence, as evidence shows that it can arise independently and even coexist with positive or negative reactions (Gasper et al., 2019). Additionally, the current investigation is devoted to exploring the effect of the participants’ sex, the effect of stimuli’s sex, and ethnicity to better understand any potential differences related to the three different developmental stages of the participants involved.

## 2. Materials and Methods

### 2.1. Participants

A total of 301 participants split into three different age groups were involved in the current study which required them to complete an online emotional decoding task. The groups included 116 children (aged 7–10, mean age = 8.56, SD = ±1.19, 60 females), 79 pre-adolescents (aged 11–13, mean age = 11.91, SD = ±.64, 46 females), and 106 adolescents (aged 14–19, mean age = 16.58, SD = ±1.72, 57 females). Regarding the age group of the adolescents taken into account for the current study, it is necessary to make a brief digression to dispel any potential doubts. In light of the scientific debate related to the precise temporal identification of the adolescent phase, it is essential to specify that the present investigation adheres to the guidelines established by the World Health Organization (WHO) and UNICEF, which define the end of this period of life as occurring at 19 years of age, which is also in line with other studies present in the literature (Patton et al., 2016; Sawyer et al., 2018; UNICEF, 2018; World Health Organization, 2019). All participants involved were of Italian nationality and subsequently of European ethnicity, and they were recruited in Campania, a region in southern Italy, mostly through several local elementary, middle, and high schools. All minors joined the study after their parents agreed to the informed consent while the adults over 18 years expressed their willingness to be involved by personally accepting the informed consent. In both cases, the informed consent was formulated according to the Italian and European laws about privacy and data protection. The research received the approval of the ethical committee of the Department of Psychology at the Università degli Studi della Campania “Luigi Vanvitelli”, with the protocol number 25/2017.

### 2.2. Stimuli

In the present study, the planned experiment involved the administration of a facial emotion recognition task. One of the major challenges in conducting this research was selecting appropriate stimuli. To our knowledge, there is a paucity of available databases containing static emotional images of children or pre-adolescents that also allow for the selection of stimuli based on different ethnicities. The Own-Age Bias in emotion recognition (ER) is well documented in the literature, indicating that children, like younger and older adults, exhibit greater accuracy in recognizing faces within their own age group compared to those from other age groups (Rhodes & Anastasi, 2012). While it would have been advantageous to investigate this effect using stimuli tailored to the three age groups examined in this study, the limited availability of datasets—such as the Child Affective Facial Expression Set (CAFE) (LoBue & Thrasher, 2015), which supports exploring multiple ethnic groups—posed a significant challenge. Consequently, the decision to use the CAFE dataset represented the best available option for the objectives of this research. Nonetheless, the chosen stimuli provide the opportunity to observe the emotional recognition abilities of the three groups involved. Specifically, this approach enables a detailed examination of the children group, whose age closely aligns with the stimuli, while also allowing for an evaluation of the ability of pre-adolescents and adolescents to recognize emotional faces representing individuals younger than themselves. The original CAFE dataset includes 1192 photographs obtained from 154 child models (90 females, 64 males) aged between 2 and 8 years. These photographs depict facial expressions corresponding to seven emotional categories: happiness, sadness, disgust, anger, fear, surprise, and neutrality. Another aim of the current study was to examine the potential effects of the sex and ethnicity of the stimuli; therefore, for each of the seven emotional categories mentioned above, eight stimuli were selected while balancing across four ethnicities (African American, Caucasian/European American, Latino, and Asian) and the two sexes. A total of 56 images were then randomly presented to each participant. To clarify the structure of the task and to ensure the study’s replicability, it is important to note that all images used are approximately 800 × 800 pixels at 96 dpi. On the screen, they are displayed at a size of 650 × 650 pixels. Furthermore, each stimulus remained visible on the screen until the participant provided a response, with a list of response options appearing simultaneously next to the target stimulus, specifically on its right.

### 2.3. Tools and Procedures

The experiment was implemented using an online study builder called Lab.js, and once fulfilled it was exported to JATOS, a tool used to generate access links. Each participant was provided with a personalized link, allowing them to access the proposed task exclusively via a laptop. As previously mentioned, although this was an online emotional recognition task, the experimenter was present during its administration. This was necessary since most participants, being minors, were recruited (with prior parental consent) primarily through local elementary, middle, and high schools. When the link was opened, the consent form for the processing of personal data appeared and the participant’s demographic data (such as sex, age, and level of education) were subsequently collected. The experiment consisted of a test session in which 6 static images were presented and an experimental session that included 56 static images. Both sessions consist of stimuli shown in randomized order. Therefore, in order to complete the emotion recognition task, each participant was asked to validate each image by selecting an emotional label from the following list of options: disgust, anger, sadness, fear, happiness, surprise, and neutrality. This list also contained an additional response option, labeled “other emotion”, to allow for further freedom in the response choice in case the participant was not satisfied with the previous options.

## 3. Results

### 3.1. Emotions Total Scores Data Analysis Description

A first data elaboration was carried out with the aim to assess participants’ ability to correctly decode the proposed emotional categories (i.e., disgust, anger, fear, sadness, happiness, surprise, and neutrality) independently from the sex and the ethnicity of the faces. When participants correctly labeled the depicted emotion, the response was coded with a score of “1”, while when the emotion was not accurately recognized the score was “0”. Each participant was shown eight stimuli for each emotional category; therefore, the means of the recognition accuracy range from zero to eight for the current analysis. Repeated measures ANOVA was performed on the collected data, considering the participants’ sex and age group (children, pre-adolescents, and adolescents, respectively) as between subjects’ variables, and total decoding scores of the proposed emotional category, considered as within subjects’ variables. The significance level was set at α < 0.05 and differences among means were assessed through Bonferroni’s post hoc tests.

#### Emotions Total Scores Results

Significant effects of the participants’ sex were observed [*F*(1, 295) = 8.841, *p* = 0.003]. Bonferroni post hoc tests revealed that this was due to female participants (mean = 6.032) who better recognized the emotional categories investigated than male participants (mean = 5.755, *p* = 0.003). No significant differences were found among the age groups of the participants involved [*F*(2, 295) = 1.453, *p* = 0.236]. Significant differences [*F*(6, 1770) = 205.213, *p* << 0.01] emerged concerning emotions recognition scores.

**Figure 1:**
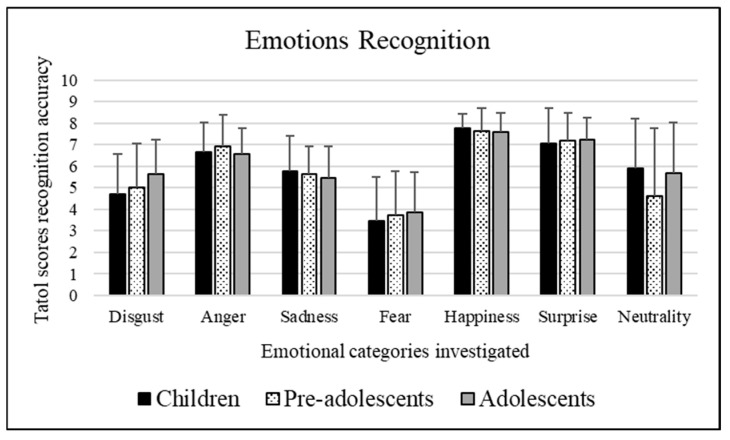
Recognition scores of children, pre-adolescents, and adolescents for each emotional category. The means illustrated on the Y axis vary between 0 and 8 and report the total decoding scores obtained by participants for each emotional category.

Bonferroni post hoc tests revealed that each emotional category significantly differed from each other: happiness (mean = 7.644), surprise (mean = 7.155), anger (mean = 6.709), sadness (mean = 5.626), neutrality (mean = 5.368), disgust (mean = 5.094), and fear (mean = 3.659), *p* < 0.01. Happiness, surprise, and anger significantly differ from all the others (*p* << 0.01) since they were the emotional categories most recognized by participants. Sadness significantly differs from the others *p* < 0.01 except neutrality (*p =* 1.000). Neutrality significantly differs from all the others (*p* << 0.01) with the exception of disgust (*p =* 1.000) and sadness (*p =* 1.000). Disgust significantly differs from all the others (*p* < 0.01) with the exception of neutrality. Lastly, fear significantly differs from all the emotional categories (*p* << 0.01) since it is the least recognized among all the others. Figure 1 shows these results.

A significant interaction emerged [*F*(12, 1770) = 4.865, *p* << 0.01] between the age groups and emotions recognition scores. Bonferroni’s post hoc tests were performed for each single factor (age groups and emotions recognition scores). These tests revealed the following:

1. Concerning age groups: Adolescents (mean = 5.627) better recognized stimuli depicting disgust than children (mean = 4.686, *p* << 0.01). Moreover, pre-adolescents (4.522) were worse at recognizing stimuli depicting neutrality than children (mean = 5.902, *p* = 0.001) and adolescents (mean = 5.679, *p* = 0.008).
2. Concerning emotions recognition scores obtained by children: Each emotional category significantly differed from each other: happiness (mean = 7.739), surprise (mean = 7.053), anger (mean = 6.652), neutrality (mean = 5.902), sadness (mean = 5.776), disgust (mean = 4.686), and fear (mean = 3.462), *p* < 0.01. Happiness significantly differs from all the others (*p* << 0.01) since it was the emotional category most recognized by participants, which was followed by surprise which significantly differs from the others *p* << 0.01, except anger (*p* = 0.224). Then, follows anger which significantly differs from the others *p* << 0.01, with the exception of surprise (as mentioned before) and neutrality (*p* = 0.140). Neutrality significantly differs from the others (*p* << 0.01), except anger (as mentioned before) and sadness (*p =* 1.000). Sadness significantly differs from all the others (*p* << 0.01) with the exception of neutrality (as already stated). Finally, disgust and fear significantly differ (*p* << 0.01) from all the other emotions as they were the least recognized. Concerning emotions recognition scores obtained by pre-adolescents: Each emotional category significantly differed from each other: happiness (mean = 7.627), surprise (mean = 7.174), anger (mean = 6.898), sadness (mean = 5.657), disgust (mean = 4.970), neutrality (mean = 4.522), and fear (mean = 3.694), *p* < 0.01. Happiness significantly differs from all the others (*p* << 0.01) since it was the emotional category most recognized by participants with the exception of surprise (*p* = 0.079). Followed by surprise which significantly differs from the others *p* << 0.01, except happiness (as already stated) and anger (*p =* 1.000). Then, follows anger which significantly differs from the others (*p* << 0.01), with the exception of surprise (as mentioned before). Sadness significantly differs from all the others (*p* < 0.05) with the exception of disgust (*p* = 0.286). Disgust significantly differs from all the others (*p* << 0.01) with the exception of sadness (as mentioned before) and neutrality (*p =* 1.000). Neutrality significantly differs from the others (*p* < 0.05), except disgust (as mentioned before) and fear (*p* = 0.623). Finally, fear appeared to be significantly different (*p* << 0.01) from all the other emotions as it was the least recognized with the exception of neutrality (as already described). Regarding emotions recognition scores obtained by adolescents: Each emotional category significantly differed from each other: happiness (mean = 7.565), surprise (mean = 7.240), anger (mean = 6.578), neutrality (mean = 5.679), disgust (mean = 5.627), sadness (mean = 5.445), and fear (mean = 3.821), *p* < 0.01. Happiness significantly differs from all the others (*p* << 0.01) since it was the emotional category most recognized by participants with the exception of surprise (*p* = 0.305). Followed by surprise which significantly differs from the others (*p* < 0.01), except happiness (as already stated). Then, anger which significantly differs from the others (*p* < 0.05). Neutrality significantly differs from the others (*p* < 0.05), except disgust (*p =* 1.000) and sadness (*p =* 1.000). Disgust and sadness differ significantly from all others (*p* << 0.01) except neutrality (*p =* 1.000) and from each other (*p =* 1.000). Lastly, fear appeared to be significantly different (*p* << 0.01) from all the other emotions since it was the least recognized among all the emotional categories explored.

To summarize the main effects, the analysis revealed that female participants were more able to recognize the explored emotional categories than male participants. Regarding the emotion recognition scores, it was clearly highlighted that the most recognized emotional categories were happiness, surprise and anger, followed by sadness, neutrality and disgust, while, interestingly, fear was the least recognized emotional category among all. Going into detail, the interaction between the variables “age groups” and “emotion recognition scores” emphasized that the emotional category of disgust was better recognized by adolescents rather than by children. Regarding neutrality, it was found that pre-adolescents obtained worse performances than children and adolescents.

### 3.2. Effects of Faces’ Ethnicity and Sex Data Analyses Description

To test the effect of the sex and the ethnicity of the displayed faces on emotion recognition accuracy, a series of ANOVA repeated measures analyses were carried out singularly for each emotional category (disgust, anger, fear, sadness, happiness, surprise, and neutrality), considering the participants’ sex and age groups (children, pre-adolescents, and adolescents, respectively) as between subjects factors and the sex and ethnicity of stimuli (Caucasian/European American, African American, Latino, and Asian) as within factors. When the emotional stimulus was correctly recognized a score of one was given, otherwise a score of zero was given. Therefore, the recognition accuracy means range from zero to one. The significance level was set at α < 0.05 and differences among means were assessed through Bonferroni’s post hoc tests.

#### 3.2.1. Disgust

**Figure 2:**
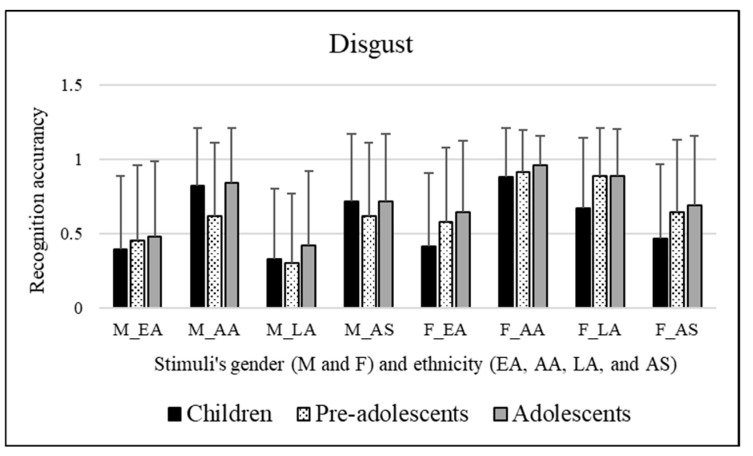
Recognition scores of children, pre-adolescents, and adolescents for disgust emotional category. Means are reported for each stimulus; first letter indicates faces’ sex, “M” for male and “F” for female, respectively, and letters after underscore refer to faces’ ethnicity (EA = Caucasian/European American, AA = African American, LA = Latino, and AS = Asian).

No significant effects were observed for participants’ sex [*F*(1, 295) = 3.852, *p* = 0.051]. Significant differences were found among the age groups of participants involved [*F*(2, 295) = 7.626, *p* = 0.001]. It emerged that the adolescents (mean = 0.703) better decoded the disgust emotion than children (mean = 0.586, *p* << 0.01). Significant effects of stimuli’s sex were observed [*F*(1, 295) = 94.338, *p* << 0.01]. Bonferroni post hoc tests revealed that this was due to female facial expressions (mean = 0.718), which were better categorized compared to male facial expressions (mean = 0.556). Significant effects of the ethnicity of stimuli were observed [*F*(3, 885) = 71.718, *p* << 0.01]. Bonferroni post hoc tests revealed that this was due to the Caucasian/European American facial expressions (mean = 0.496), which were more poorly recognized compared to African American (mean = 0.834, *p* << 0.01), Latino (mean = 0.582, *p* = 0.003), and Asian (mean = 0.635, *p* << 0.01) facial expressions. Moreover, African American facial expressions (mean = 0.834) were better decoded than Latino (mean = 0.582, *p* << 0.01) and Asian (mean = 0.635, *p* << 0.01) facial expressions. Figure 2 illustrates these results.

A significant interaction emerged [*F*(2, 295) = 15.040, *p* << 0.01] between the participants’ age groups and stimuli’s sex. Bonferroni’s post hoc tests were performed for each single factor (participants’ age groups and stimuli’s sex). These tests revealed the following:

1. Concerning participants’ age groups: Adolescents (mean = 0.612) better decoded male facial expressions than pre-adolescents (mean = 0.491, *p* = 0.014). Moreover, children (mean = 0.607) were worse at recognizing female facial expressions than adolescents (mean = 0.795, *p* << 0.01) and pre-adolescents (mean = 0.751, *p* << 0.01).
2. Concerning stimuli’s sex: Pre-adolescents better decoded female stimuli (mean = 0.751) compared to male stimuli (mean = 0.491, *p* << 0.01). Similarly, adolescents better decoded female stimuli (mean = 0.795) compared to male stimuli (mean = 0.612, *p* << 0.01).

A significant interaction emerged [*F*(6, 885) = 2.507, *p* = 0.021] between participants’ age groups and stimuli’s ethnicity. Bonferroni’s post hoc tests were performed for each single factor (participants’ age groups and stimuli’s ethnicity). These tests revealed the following:

1. Concerning participants’ age groups: Caucasian/European American facial expressions were better decoded by adolescents (mean = 0.562) than children (mean = 0.405, *p* = 0.006). Adolescents (mean = 0.898) better recognized African American facial expressions than pre-adolescents (mean = 0.756, *p* = 0.003). Moreover, adolescents (mean = 0.655) better recognized Latino facial expressions than children (mean = 0.500, *p* = 0.001).
2. Concerning stimuli’s ethnicity: Children better decoded African American facial expressions (mean = 0.849, *p* << 0.01) compared to Caucasian/European American (mean = 0.405), Latino (mean = 0.500), and Asian facial expressions (mean = 0.590). Regarding pre-adolescents, they better decoded African American facial expressions (mean = 0.756) compared to Caucasian/European American (mean = 0.521, *p* << 0.01), Latino (mean = 0.592, *p* = 0.001), and Asian facial expressions (mean = 0.616, *p* = 0.008). Lastly, concerning adolescents, they better decoded African American facial expressions (mean = 0.898, *p* << 0.01) compared to Caucasian/European American (mean = 0.562), Latino (mean = 0.655), and Asian facial expressions (mean = 0.699). In addition, adolescents better decoded Asian facial expressions (mean = 0.699) compared to Caucasian/European American facial expressions (mean = 0.562, *p* = 0.010).

A significant interaction emerged [*F*(3, 885) = 46.843, *p* << 0.01] between stimuli’s sex and ethnicity. Bonferroni’s post hoc tests were performed for each single factor (stimuli’s sex and ethnicity). These tests revealed the following:

1. Concerning stimuli’s sex: Caucasian/European American facial expressions were better decoded when conveyed by female stimuli (mean = 0.548) compared to male stimuli (mean = 0.443, *p* = 0.006). Similarly, African American facial expressions were better decoded when conveyed by female stimuli (mean = 0.917) compared to male stimuli (mean = 0.752, *p* << 0.01). Interestingly, Latino facial expressions were also better decoded when conveyed by female stimuli (mean = 0.814) compared to male stimuli (mean = 0.351, *p* << 0.01). Conversely, Asian facial expressions were better decoded when conveyed by male stimuli (mean = 0.679) compared to female stimuli (mean = 0.591, *p* = 0.015).
2. Concerning stimuli’s ethnicity: Male Caucasian/European American facial expressions (mean = 0.443, *p* << 0.01) were more poorly decoded compared to male African American (mean = 0.752) and male Asian facial expressions (mean = 0.679). Moreover, male Latino facial expressions (mean = 0.351, *p* << 0.01) were more poorly decoded than male African American (mean = 0.752) and male Asian facial expressions (mean = 0.679). Female African American facial expressions (mean = 0.917, *p* << 0.01) were better decoded compared to female Caucasian/European American (mean = 0.548), female Latino (mean = 0.814), and female Asian facial expressions (mean = 0.591). Moreover, female Latino facial expressions (mean = 0.814, *p* << 0.01) were better decoded than female Caucasian/European American (mean = 0.548) and female Asian facial expressions (mean = 0.591).

In summary, the outcomes revealed that adolescents better decoded the emotional category of disgust than children. The female facial expressions were categorized more accurately than male ones. Regarding the variable ethnicity of the stimuli, it was found that AA facial expressions were better decoded than LA and AS facial expressions, while EA facial expressions were the least recognized among all the ethnicities examined.

Going deeper, the interactions between the variables under study added the following information: adolescents were more accurate in recognizing male facial expressions than pre-adolescents. On the other hand, children were worse at recognizing female facial expressions compared to adolescents and pre-adolescents. Pre-adolescents and adolescents better decoded female stimuli than male ones. Adolescents recognized AA facial expressions better than pre-adolescents. Furthermore, adolescents better recognized EA and LA facial expressions than children. All the three aged groups were more capable of recognizing AA facial expressions than EA, LA, and AS facial expressions. Furthermore, adolescents decoded AS facial expressions better than EA expressions. EA, AA, and LA facial expressions were better decoded when portrayed by female stimuli than by male stimuli. On the contrary, AS facial expressions were better decoded when portrayed by male stimuli than by female stimuli. Male EA and LA facial expressions were more poorly decoded than male AA and AS facial expressions. Female AA facial expressions were better decoded than female EA, LA, and AS facial expressions. Additionally, female LA expressions were better decoded than female EA and AS facial expressions.

#### 3.2.2. Anger

**Figure 3:**
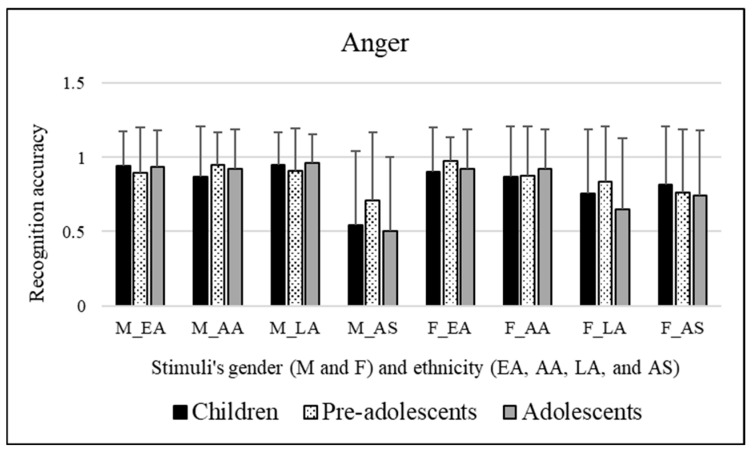
Recognition scores of children, pre-adolescents, and adolescents for anger emotional category. Means are reported for each stimulus; first letter indicates faces’ sex, “M” for male and “F” for female, respectively, and letters after underscore refer to faces’ ethnicity (EA = Caucasian/European American, AA = African American, LA = Latino, and AS = Asian).

Significant effects were not observed for the participants’ sex [*F*(1, 295) = 0.008, *p* = 0.928] or for the age groups of participants involved [*F*(2, 295) = 1.322, *p* = 0.268]. No significant effects of stimuli’s sex were observed [*F*(1, 295) = 0.026, *p* = 0.871]. Significant effects of the ethnicity of stimuli were observed [*F*(3, 885) = 7.017, *p* << 0.01]. Bonferroni post hoc tests revealed that this was due to Asian facial expressions (mean = 0.680, *p* << 0.01), which were more poorly recognized compared to Caucasian/European American (mean = 0.928), African American (mean = 0.902), and Latino (mean = 0.844) facial expressions. Moreover, Latino facial expressions (mean = 0.844) were more poorly decoded than Caucasian/European American (mean = 0.928, *p* << 0.01) and African American (mean = 0.902, *p* = 0.004) facial expressions. Figure 3 shows these results.

A significant interaction emerged [*F*(3, 885) = 35.583, *p* << 0.01] between stimuli’s sex and ethnicity. Bonferroni’s post hoc tests were performed for each single factor (stimuli’s sex and ethnicity). These tests revealed the following:

1. Concerning stimuli’s sex: Latino facial expressions were better decoded when conveyed by male stimuli (mean = 0.941) compared to female stimuli (mean = 0.746, *p* << 0.01). Conversely, Asian facial expressions were better decoded when conveyed by female stimuli (mean = 0.778) compared to male stimuli (mean = 0.583, *p* << 0.01).
2. Concerning stimuli’s ethnicity: Male Asian facial expressions (mean = 0.583, *p* << 0.01) were poorly decoded compared to male Caucasian/European American (mean = 0.922), male African American (mean = 0.914), and male Latino facial expressions (mean = 0.941). Moreover, female Caucasian/European American facial expressions (mean = 0.935, *p* << 0.01) were better decoded compared to female Latino (mean = 0.746) and female Asian (mean = 0.778) facial expressions. Similarly, female African American facial expressions (mean = 0.891) were better decoded compared to female Latino (mean = 0.746, *p* << 0.01) and female Asian (mean = 0.778, *p* = 0.001) facial expressions.

To resume, the analysis revealed that AS facial expressions were poorly recognized compared to EA, AA, and LA facial expressions. In addition, LA facial expressions were more poorly decoded than EA and AA facial expressions.

Interestingly, the interaction that emerged between stimuli’s sex and ethnicity variables highlighted that LA facial expressions were better decoded when conveyed by male stimuli compared to female stimuli, while AS facial expressions were better decoded when conveyed by female stimuli compared to male stimuli. Male AS facial expressions were more poorly decoded compared to male EA, AA, and LA facial expressions, while female EA and AA facial expressions were better decoded compared to female LA and AS facial expressions.

#### 3.2.3. Sadness

**Figure 4:**
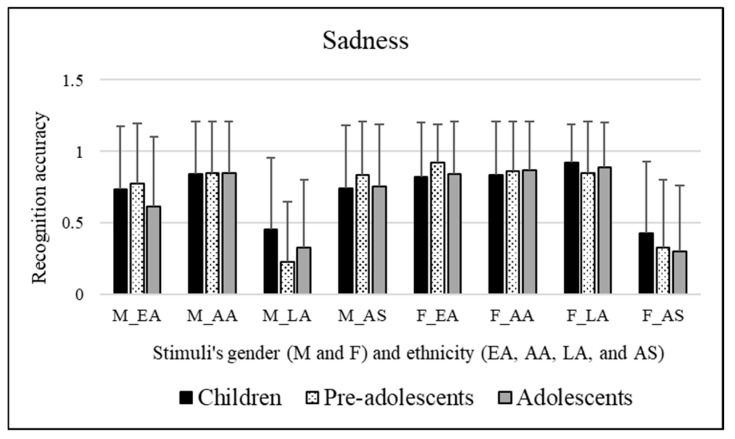
Recognition scores of children, pre-adolescents, and adolescents for sadness emotional category. Means are reported for each stimulus; first letter indicates faces’ sex, “M” for male and “F” for female, respectively, and letters after underscore refer to faces’ ethnicity (EA = Caucasian/European American, AA = African American, LA = Latino, and AS = Asian).

No significant effects were observed for the participants’ sex [*F*(1, 295) = 0.396, *p* = 0.530] or for the age groups of the participants involved [*F*(2, 295) = 1.409, *p* = 0.246]. Significant effects of the stimuli’s sex were observed [*F*(1, 295) = 22.304, *p* << 0.01]. Bonferroni post hoc tests revealed that this was due to female facial expressions (mean = 0.740), which were better categorized compared to male facial expressions (mean = 0.667). Significant effects of the ethnicity of stimuli were observed [*F*(3, 885) = 72.723, *p* << 0.01]. Bonferroni post hoc tests revealed that this was due to African American facial expressions (mean = 0.852), which were better recognized compared to Caucasian/European American (mean = 0.783, *p* = 0.008), Latino (mean = 0.610, *p* << 0.01), and Asian (mean = 0.567, *p* << 0.01) facial expressions. Moreover, Caucasian/European American facial expressions (mean = 0.783, *p* << 0.01) were better decoded than Latino (mean = 0.610) and Asian (mean = 0.567) facial expressions. These results are shown in Figure 4.

A significant interaction emerged [*F*(6, 885) = 3.924, *p* = 0.001] between the participants’ age groups and stimuli’s ethnicity. Bonferroni’s post hoc tests were performed for each single factor (participants’ age groups and stimuli’s ethnicity). These tests revealed the following:

1. Concerning participants’ age groups: Caucasian/European American facial expressions were better decoded by pre-adolescents (mean = 0.850) than adolescents (mean = 0.726, *p* = 0.024). Children (mean = 0.688) better recognized Latino facial expressions than pre-adolescents (mean = 0.530, *p* = 0.001).
2. Concerning stimuli’s ethnicity: Children were worse at decoding Asian facial expressions (mean = 0.585) compared to Caucasian/European American expressions (mean = 0.774, *p* << 0.01). Moreover, they better decoded African American facial expressions (mean = 0.840, *p* << 0.01) compared to Latino (mean = 0.688) and Asian (mean = 0.585) facial expressions. Regarding pre-adolescents, they better decoded Caucasian/European American facial expressions (mean = 0.850, *p* << 0.01) compared to Latino (mean = 0.530) and Asian facial expressions (mean = 0.590). Moreover, they better decoded African American facial expressions (mean = 0.858, *p* << 0.01) compared to Latino (mean = 0.530) and Asian facial expressions (mean = 0.590). Lastly, concerning adolescents, they better decoded African American facial expressions (mean = 0.858) compared to Caucasian/European American (mean = 0.726, *p* = 0.001), Latino (mean = 0.611, *p* << 0.01), and Asian facial expressions (mean = 0.527, *p* << 0.01). In addition, adolescents better decoded Caucasian/European American facial expressions (mean = 0.726) compared to Latino (mean = 0.611, *p* = 0.017) and Asian facial expressions (mean = 0.527, *p* << 0.01).

A significant interaction emerged [*F*(3, 885) = 151.518, *p* << 0.01] between the stimuli’s sex and ethnicity. Bonferroni’s post hoc tests were performed for each single factor (stimuli’s sex and ethnicity). These tests revealed the following:

1. Concerning stimuli’s sex: Caucasian/European American facial expressions were better decoded when conveyed by female stimuli (mean = 0.861) compared to male stimuli (mean = 0.706, *p* << 0.01). Similarly, Latino facial expressions were better decoded when conveyed by female stimuli (mean = 0.885) compared to male stimuli (mean = 0.334, *p* << 0.01). Conversely, Asian facial expressions were better decoded when conveyed by male stimuli (mean = 0.778) compared to female stimuli (mean = 0.357, *p* << 0.01).
2. Concerning stimuli’s ethnicity: Male Latino facial expressions (mean = 0.334, *p* << 0.01) were poorly decoded compared to male Caucasian/European American (mean = 0.706), male African American (mean = 0.849), and male Asian facial expressions (mean = 0.778). Moreover, male African American facial expressions (mean = 0.849, *p* << 0.01) were better decoded compared to male Caucasian/European American (mean = 0.706) facial expressions. Female Asian facial expressions (mean = 0.357, *p* << 0.01) were poorly decoded compared to female Caucasian/European American (mean = 0.861), female African American (mean = 0.855), and Latino facial expressions (mean = 0.855).

To sum up, female facial expressions were better categorized compared to male ones. AA facial expressions were better recognized compared to EA, LA, and AS facial expressions. Moreover, EA facial expressions were better decoded than LA and AS facial expressions.

The interactions between the variables examined have shown the following: EA facial expressions were better decoded by pre-adolescents rather than adolescents, while children demonstrated to be more capable in decoding LA facial expressions than pre-adolescents. Specifically, children were worse at decoding AS facial expressions compared to EA facial expressions, while they better decoded AA facial expressions compared to LA and AS facial expressions. Pre-adolescents were better at decoding EA and AA facial expressions compared to LA and AS facial expressions. Lastly, adolescents better decoded AA facial expressions compared to the other three ethnicities involved, and they were more accurate in decoding EA facial expressions compared to LA and AS facial expressions. Female EA and LA facial expressions were better decoded compared to male EA and LA stimuli. Regarding AS facial expressions, they were better decoded when portrayed by male stimuli compared to female stimuli. Male LA facial expressions were more poorly decoded compared to male EA, AA, and AS expressions. Moreover, male AA facial expressions were better decoded compared to male EA facial expressions. Female AS facial expressions were poorly decoded compared to female EA, AA, and LA facial expressions.

#### 3.2.4. Fear

**Figure 5:**
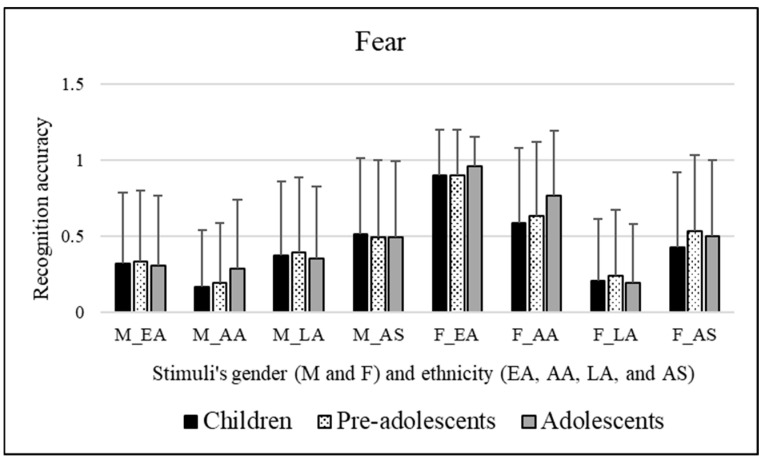
Recognition scores of children, pre-adolescents, and adolescents for fear emotional category. Means are reported for each stimulus; first letter indicates faces’ sex, “M” for male and “F” for female, respectively, and letters after underscore refer to faces’ ethnicity (EA = Caucasian/European American, AA = African American, LA = Latino, and AS = Asian).

Slightly significant effects were observed for the participants’ sex [*F*(1, 295) = 3.924, *p* = 0.049]. Bonferroni post hoc tests revealed that this was due to the female participants (mean = 0.486), who better recognized the emotional category of fear than male participants (mean = 0.429). No significant effect was observed for the age groups of participants involved [*F*(2, 295) = 0.933, *p* = 0.394]. Significant effects of the stimuli’s sex were observed [*F*(1, 295) = 186.124, *p* << 0.01]. Bonferroni post hoc tests revealed that this was due to female facial expressions (mean = 0.565), which were better categorized compared to male facial expressions (mean = 0.349). Significant effects of the ethnicity of stimuli were observed [*F*(3, 885) = 63.291, *p* << 0.01]. Bonferroni post hoc tests revealed that this was due to Caucasian/European American facial expressions (mean = 0.615, *p* << 0.01), which were better recognized compared to African American (mean = 0.433), Latino (mean = 0.291), and Asian (mean = 0.490) facial expressions. Moreover, Latino facial expressions (mean = 0.291, *p* << 0.01) were more poorly decoded than African American (mean = 0.433) and Asian (mean = 0.490) facial expressions. Figure 5 reports these results.

A significant interaction emerged [*F*(6, 885) = 2.229, *p* = 0.038] between the participants’ age groups and stimuli’s ethnicity. Bonferroni’s post hoc tests were performed for each single factor (participants’ age groups and stimuli’s ethnicity). These tests revealed the following:

1. Concerning participants’ age groups: African American facial expressions were better decoded by adolescents (mean = 0.520) than children (mean = 0.374, *p* = 0.004).
2. Concerning stimuli’s ethnicity: Children better decoded Caucasian/European American facial expressions (mean = 0.606) compared to African American (mean = 0.374, *p* << 0.01), Latino (mean = 0.288, *p* << 0.01), and Asian (mean = 0.464, *p* = 0.003) facial expressions. Moreover, they were worse at decoding Latino facial expressions (mean = 0.288) compared to Asian (mean = 0.464, *p* << 0.01) facial expressions. Pre-adolescents better decoded Caucasian/European American facial expressions (mean = 0.609) compared to African American (mean = 0.405, *p* << 0.01) and Latino (mean = 0.319, *p* << 0.01) facial expressions. Moreover, they poorly decoded Latino facial expressions (mean = 0.319) compared to Asian (mean = 0.515, *p* << 0.01) facial expressions. Lastly, adolescents better decoded Caucasian/European American facial expressions (mean = 0.632) compared to African American (mean = 0.520, *p* = 0.009), Latino (mean = 0.266, *p* << 0.01) and Asian (mean = 0.491, *p* = 0.006) facial expressions. Moreover, they were worse at decoding Latino facial expressions (mean = 0.266, *p* << 0.01) compared to African American (mean = 0.520) and Asian (mean = 0.491) facial expressions.

A significant interaction emerged [*F*(3, 885) = 127.457, *p* << 0.01] between the stimuli’s sex and ethnicity. Bonferroni’s post hoc tests were performed for each single factor (stimuli’s sex and ethnicity). These tests revealed the following:

1. Concerning stimuli’s sex: Caucasian/European American facial expressions were better decoded when conveyed by female stimuli (mean = 0.914) compared to male stimuli (mean = 0.316, *p* << 0.01). Similarly, African American facial expressions were better decoded when conveyed by female stimuli (mean = 0.656) compared to male stimuli (mean = 0.210, *p* << 0.01). Conversely, Latino facial expressions were better decoded when conveyed by male stimuli (mean = 0.373) compared to female stimuli (mean = 0.209, *p* << 0.01).
2. Concerning stimuli’s ethnicity: Male African American facial expressions (mean = 0.210) were poorly decoded compared to male Caucasian/European American (mean = 0.316, *p* = 0.009), male Latino (mean = 0.373, *p* << 0.01), and male Asian facial expressions (mean = 0.498, *p* << 0.01). Moreover, male Asian facial expressions (mean = 0.498) were better decoded compared to male Caucasian/European American (mean = 0.316, *p* << 0.01) and male Latino (mean = 0.373, *p* = 0.001) facial expressions. Female Caucasian/European American facial expressions (mean = 0.914, *p* << 0.01) were better decoded compared to female African American (mean = 0.656), female Latino (mean = 0.209), and female Asian facial expressions (mean = 0.482). Moreover, female African American facial expressions (mean = 0.656, *p* << 0.01) were better decoded compared to female Latino (mean = 0.209) and female Asian facial expressions (mean = 0.482). Conversely, female Latino (mean = 0.209) were more poorly decoded than female Asian (mean = 0.482, *p* << 0.01) facial expressions.

To resume, female participants demonstrated that they were more capable in recognizing the emotional category of fear than male participants. Female facial expressions were better categorized compared to male facial expressions. EA facial expressions were better recognized compared to AA, LA, and AS facial expressions. Conversely, LA facial expressions were more poorly decoded than AA and AS facial expressions.

The interactions between the variables provided the following information: AA facial expressions were better decoded by adolescents than children. Going into detail, concerning the differences among the participants’ groups involved, children better decoded EA facial expressions compared to the other three ethnicities investigated. Moreover, they more poorly decoded LA facial expressions compared to AS facial expressions. Regarding pre-adolescents, they better decoded EA facial expressions compared to AA and LA facial expressions. Moreover, as occurred for children, pre-adolescents were also worse at decoding LA facial expressions compared to AS facial expressions. Lastly, as observed for children, adolescents also better decoded EA facial expressions compared to the other three ethnicities investigated. Furthermore, they more poorly decoded LA facial expressions compared to AA and AS facial expressions. Regarding the stimuli’s sex, EA and AA were better decoded when portrayed by female stimuli than male ones. On the contrary, LA facial expressions were better decoded when portrayed by male stimuli than female ones. Male AA facial expressions poorly decoded compared to male EA, LA, and AS facial expressions. Moreover, male AS facial expressions were better decoded compared to male EA and LA facial expressions. Female EA facial expressions were better decoded compared to female AA, LA, and AS facial expressions. In addition, female AA facial expressions were better decoded compared to female LA and AS facial expressions. Conversely, female LA were more poorly decoded than female AS facial expressions.

#### 3.2.5. Surprise

**Figure 6:**
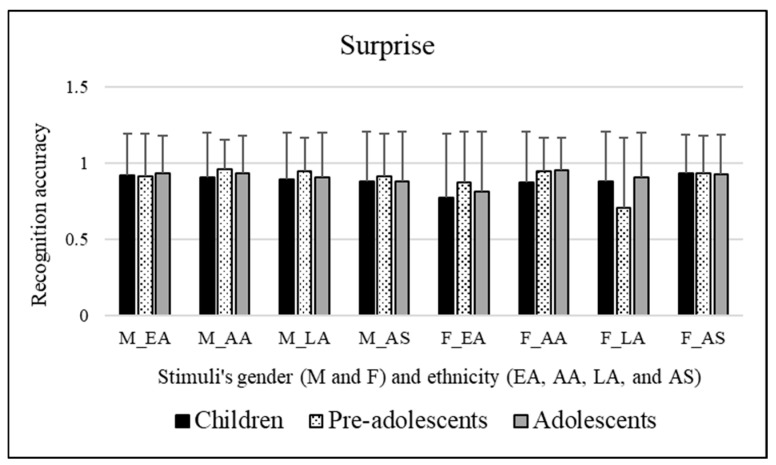
Recognition scores of children, pre-adolescents, and adolescents for surprise emotional category. Means are reported for each stimulus; first letter indicates faces’ sex, “M” for male and “F” for female, respectively, and letters after underscore refer to faces’ ethnicity (EA = Caucasian/European American, AA = African American, LA = Latino, and AS = Asian).

No significant effects of the participants’ sex [*F*(1, 295) = 3.886, *p* = 0.050] or significant effects for the age groups of participants involved [*F*(2, 295) = 0.556, *p* = 0.574] were observed. Significant effects of the stimuli’s sex were observed [*F*(1, 295) = 13.375, *p* << 0.01]. Bonferroni post hoc tests revealed that this was due to male facial expressions (mean = 0.915), which were better categorized compared to female facial expressions (mean = 0.874). Significant effects of the ethnicity of stimuli were observed [*F*(3, 885) = 6.274, *p* << 0.01]. Bonferroni post hoc tests revealed that this was due to African American facial expressions (mean = 0.928), which were better recognized compared to Caucasian/European American (mean = 0.870, *p* = 0.003) and Latino (mean = 0.871, *p* = 0.001) facial expressions. Figure 6 reveals these results.

A significant interaction emerged [*F*(6, 885) = 2.488, *p* = 0.022] between the participants’ age groups and stimuli’s ethnicity. Bonferroni’s post hoc tests were performed for each single factor (participants’ age groups and stimuli’s ethnicity). These tests revealed the following:

1. Concerning participants’ age groups: Since Bonferroni post hoc analysis tends to be very restrictive in this case the main effect disappeared. Therefore, there were no significant differences among the three levels of age groups investigated in decoding the different facial expressions of the ethnicities proposed.
2. Concerning stimuli’s ethnicity: Pre-adolescents were worse at decoding Latino facial expressions (mean = 0.823) compared to African American (mean = 0.953, *p* << 0.01) and Asian (mean = 0.920, *p* = 0.013) facial expressions. Adolescents better decoded African American facial expressions (mean = 0.944) compared to Caucasian/European American (mean = 0.872, *p* = 0.049) facial expressions.

A significant interaction emerged [*F*(3, 885) = 9.199, *p* << 0.01] between the stimuli’s sex and ethnicity. Bonferroni’s post hoc tests were performed for each single factor (stimuli’s sex and ethnicity). These tests revealed the following:

1. Concerning stimuli’s sex: Caucasian/European American facial expressions were better decoded when conveyed by male stimuli (mean = 0.922) compared to female stimuli (mean = 0.819, *p* << 0.01). Similarly, Latino facial expressions were better decoded when conveyed by male stimuli (mean = 0.917) compared to female stimuli (mean = 0.826, *p* << 0.01).
2. Concerning stimuli’s ethnicity: Female Caucasian/European American facial expressions (mean = 0.819, *p* << 0.01) were poorly decoded compared to female African American (mean = 0.925) and female Asian facial expressions (mean = 0.925). Moreover, female Latino (mean = 0.826, *p* << 0.01) facial expressions were more poorly decoded than female African American (mean = 0.925) and female Asian facial expressions (mean = 0.925).

In summary, male facial expressions were better categorized compared to female ones. AA facial expressions were better recognized compared to EA and LA facial expressions.

Going deeper, the analysis on the interactions revealed that pre-adolescents were worse at decoding LA facial expressions compared to AA and AS facial expressions. Adolescents better decoded AA facial expressions compared to EA facial expressions. Furthermore, EA and La facial expressions were better decoded when transmitted by male stimuli rather than female stimuli. Female EA and LA facial expressions were poorly decoded compared to female AA and AS facial expressions.

#### 3.2.6. Happiness

**Figure 7:**
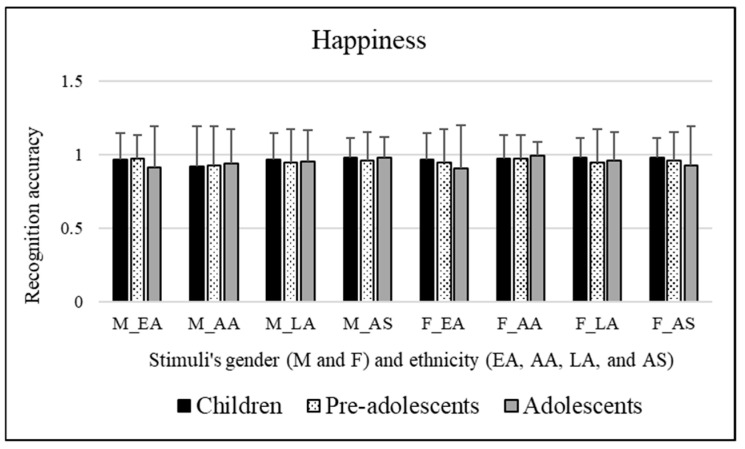
Recognition scores of children, pre-adolescents, and adolescents for happiness emotional category. Means are reported for each stimulus; first letter indicates faces’ sex, “M” for male and “F” for female, respectively, and letters after underscore refer to faces’ ethnicity (EA = Caucasian/European American, AA = African American, LA = Latino, and AS = Asian).

Significant effects of the participants’ sex were observed [*F*(1, 295) = 4.581, *p* = 0.033]. Bonferroni post hoc tests revealed that this was due to female participants (mean = 0.969), who better recognized the emotional category of happiness than male participants (mean = 0.942). No significant effects were observed for the age groups of the participants involved [*F*(2, 295) = 1.143, *p* = 0.320]. Neither significant effects of the stimuli’s sex [*F*(1, 295) = 1.532 *p* = 0.217] or significant effects of the ethnicity of stimuli were observed [*F*(3,885) = 1.139, *p* = 0.332]. Figure 7 shows these results.

A significant interaction emerged [*F*(3, 885) = 4.604, *p* = 0.003] between the stimuli’s sex and ethnicity. Bonferroni’s post hoc tests were performed for each single factor (stimuli’s sex and ethnicity). These tests revealed the following:

1. Concerning stimuli’s sex: African American facial expressions were better decoded when conveyed by female stimuli (mean = 0.979) compared to male stimuli (mean = 0.926, *p* = 0.001).
2. Concerning stimuli’s ethnicity: Male Asian facial expressions (mean = 0.975, *p* = 0.011) were better decoded compared to male African American (mean = 0.926) facial expressions. Moreover, female African American (mean = 0.979, *p* = 0.049) expressions were better decoded than female Caucasian/European American facial expressions (mean = 0.941).

To sum up, female participants were revealed to be more accurate in decoding the emotional category of happiness than male participants.

The analysis on the interaction highlighted that AA facial expressions were better decoded when conveyed by female stimuli compared to male ones. Male AS facial expressions were better decoded compared to male AA facial expressions. Moreover, female AA facial expressions were better decoded than female EA facial expressions.

#### 3.2.7. Neutrality

**Figure 8:**
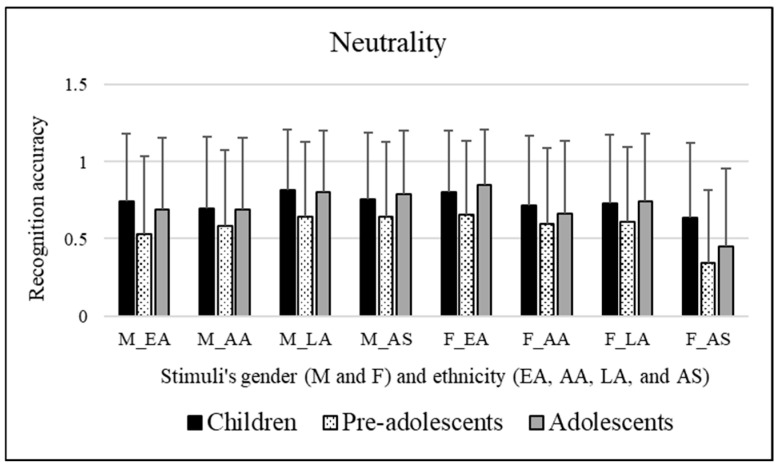
Recognition scores of children, pre-adolescents, and adolescents for neutrality emotional category. Means are reported for each stimulus; first letter indicates faces’ sex, “M” for male and “F” for female, respectively, and letters after underscore refer to faces’ ethnicity (EA = Caucasian/European American, AA = African American, LA = Latino, and AS = Asian).

No significant effects of the participants’ sex were observed [*F*(1, 295) = 1.812, *p* = 0.179]. Significant differences were found for the age groups of participants involved [*F*(2, 295) = 7.273, *p* = 0.001]. It emerged that the pre-adolescents (mean = 0.565) were worse at decoding neutrality than children (mean = 0.738, *p* = 0.001) and adolescents (mean = 0.710, *p* = 0.008). Significant effects of the stimuli’s sex were observed [*F*(1, 295) = 15.889, *p* << 0.01]. Bonferroni post hoc tests revealed that this was due to male facial expressions (mean = 0.697), which were better categorized compared to female facial expressions (mean = 0.645). Significant effects of the ethnicity of stimuli were observed [*F*(3, 885) = 14.459, *p* << 0.01]. Bonferroni post hoc tests revealed that this was due to Asian facial expressions (mean = 0.601), which were poorly recognized compared to Caucasian/European American (mean = 0.709, *p* << 0.01) and Latino (mean = 0.721, *p* << 0.01) facial expressions. Moreover, Latino facial expressions (mean = 0.721) were better decoded than African American (mean = 0.653, *p* = 0.004) facial expressions. Figure 8 exemplifies these results.

A significant interaction emerged [*F*(3, 885) = 28.824, *p* << 0.01] between the stimuli’s sex and ethnicity. Bonferroni’s post hoc tests were performed for each single factor (stimuli’s sex and ethnicity). These tests revealed the following:

1. Concerning stimuli’s sex: Caucasian/European American facial expressions were better decoded when conveyed by female stimuli (mean = 0.765) compared to male stimuli (mean = 0.654, *p* << 0.01). Conversely, Latino facial expressions were better decoded when conveyed by male stimuli (mean = 0.755) compared to female stimuli (mean = 0.687, *p* = 0.006). Similarly, Asian facial expressions were better decoded when conveyed by male stimuli (mean = 0.727) compared to female stimuli (mean = 0.475, *p* << 0.01).
2. Concerning stimuli’s ethnicity: Male Latino facial expressions (mean = 0.755) were better decoded compared to male Caucasian/European American (mean = 0.654, *p* = 0.003) and male African American facial expressions (mean = 0.652, *p* = 0.001). Moreover, male Asian facial expressions (mean = 0.727) were better decoded compared to male African American facial expressions (mean = 0.652, *p* = 0.025). Female Asian facial expressions (mean = 0.475, *p* << 0.01) were more poorly decoded compared to female Caucasian/European American (mean = 0.765), female African American (mean = 0.654), and female Latino (mean = 0.687) facial expressions. Moreover, female Caucasian/European American facial expressions (mean = 0.765) were better decoded compared to female African American (mean = 0.654, *p* = 0.001) and female Latino (mean = 0.687, *p* = 0.016) facial expressions.

To resume, pre-adolescents were worse at decoding neutrality than children and adolescents. Male facial expressions were better categorized compared to female facial expressions. AS facial expressions were poorly recognized compared to EA and LA facial expressions. On the contrary, LA facial expressions were better decoded than AA facial expressions.

From the analysis conducted on the interaction it emerged that EA facial expressions were better decoded when transmitted by female stimuli compared to male ones. Conversely, LA and AS facial expressions were better decoded when transmitted by male stimuli compared to female ones. Male LA facial expressions were better decoded compared to male EA and AA facial expressions. Moreover, male AS facial expressions were better decoded compared to male AA ones. Female AS facial expressions were more poorly decoded than female EA, AA, and LA facial expressions. On the contrary, female EA facial expressions were better decoded compared to female AA and LA facial expressions.

### 3.3. The Emotion Recognition Accuracy in Percentage Values Computed for Children, Pre-Adolescents, and Adolescents Divided Between Male and Female Participants

This descriptive section summarizes the percentage values of emotion recognition accuracy computed for children, pre-adolescents, and adolescents, as reported in Table 1. In regard to the emotional category of disgust, it emerged that 69.2% of female adolescents were better at recognizing the static stimuli proposed compared to the other groups, and was followed by the 59.8% achieved by female children in line with the statistical analysis described above and performed for disgust which highlighted an advantage in the disgust decoding of adolescents compared to children. Lower recognition percentage values are recorded for both male children (53.5%) and male adolescents (55.4%), proving to be quite similar. Curiously, however, concerning pre-adolescents’ emotion recognition, the percentage values of female pre-adolescents (12.5%) are lower than those obtained for male pre-adolescents (31.9%), thus revealing that male pre-adolescents are more skilled than female pre-adolescents in recognizing facial expressions of disgust. Nonetheless, pre-adolescents of both sexes appear to be less accurate than other groups in correctly recognizing disgust, underlining an interesting decrease in accuracy in this intermediate developmental stage compared to both the previous stage and the following one. Decidedly higher recognition percentage values are found for the emotional category of anger, which from static analyses proves to be among the best recognized emotions. In this case, however, both male (76.5%) and female (84.4%) children labeled anger better than the other groups (male adolescents = 68.8%; female adolescents = 76.3%; male pre-adolescents = 46.9%; and female pre-adolescents = 11.5%). Also in this case, a decrease in the accuracy of recognition by pre-adolescents is observed, mostly affecting the female subgroup. The same trend is also observed for the emotional category of sadness with generally lower percentage values than anger. Both male (64.4%) and female (75.4%) children more accurately recognized sadness than the other groups (male adolescents = 55.8%; female adolescents = 64.4%; male pre-adolescents = 39.4%; and female pre-adolescents = 13.3%). Also in this case, a decrease in the accuracy of the recognition by pre-adolescents is observed, mostly influencing the female participants. In regard to the emotional category of fear, both from the percentage values presented in Table 1 and from the statistical analysis, it was found to be the worst recognized facial expression. Going into more detail, the percentage values indicate a better recognition by adolescents (females = 48.3%; males = 36.5%) compared to both children (females = 47.7%; males = 36.3%) and pre-adolescents who are still in last position, and also, in this case, within the age group there are differences between males (24.8%) and females (12.5%), which reveal a substantial decrease on the part of female pre-adolescents. The emotional category of happiness follows, which turned out to be the best recognized both by the statistical analyses and percentage values reported in Table 1. In this case, children of both sexes, with a slight female advantage (females = 97.7%; males = 89.4%), obtained better recognition performances than adolescents (females = 91.5%; males = 75.8%), and were followed finally by pre-adolescents (females = 12.5%; males = 51.7%), where the female disadvantage is most overwhelming. Concerning surprise, which appears to be the second best recognized emotional category after happiness, the recognition percentage values indicate the same trend observed for happiness, revealing that children of both sexes (females = 90.8%; males = 79.8%) obtained better recognition performances (with a slight female increase) than adolescents (females = 86.9%; males = 73.1%), and they are consequently followed by pre-adolescents (females = 9.4%; males = 48.1%) with the usual female decrease. From a statistical point of view, the pre-adolescents involved in the present study obtained worse performances in recognizing neutrality than adolescents and children, which is in line with the accuracy percentage values reported in Table 1. In fact, children (females = 74.8%; males = 67.9%) and adolescents (females = 67.5%; males = 57.9%) seem to better recognize neutrality than pre-adolescents. Specifically, observing both subgroups, a better recognition of male pre-adolescents (27.5%) than female pre-adolescents (12.5%) emerged, which is in line with the considerations relating to other emotional categories previously described.

## 4. Discussion

This research aimed to analyze the effects of participants’ sex and age, as well as the impact of variables such as the ethnicity and sex of the stimuli, through an emotional recognition task of static facial expressions concerning the following emotional categories: happiness, sadness, anger, fear, disgust, surprise, and neutrality. Since it is consistently reported in the literature that emotional recognition skills are refined from childhood to adolescence (Gao et al., 2010; Rodger et al., 2015; Ewing et al., 2017), three groups of participants belonging to the three different developmental stages, namely childhood, preadolescence, and adolescence, were recruited to explore differences in emotional identification processes, in an attempt to enrich the existing literature on the topic. Regarding the analysis performed on the total emotion scores independently from the ethnicity and sex of the stimuli, the analysis revealed that the female participants recognized the investigated emotional categories better than the male participants. Outcomes of the current study appear to be in line with the evidence showing a female advantage in emotion recognition (Montirosso et al., 2010; Olderbak et al., 2019), which is in contrast with a whole line of research that does not highlight sex differences linked to emotional recognition skills. This line of research states that this gap attenuates during preadolescence as, for example, can be seen in the emotional category of sadness, and through an initial disadvantage it is possible that male pre-adolescents achieve an improvement of these capacities after 11 years and are able to match females’ ability to accurately label and recognize emotions (Mancini et al., 2013; Mancini et al., 2018). The present study demonstrated that among the facial emotional expressions investigated, the most recognized are happiness, surprise, and anger, followed by sadness, neutrality, and disgust. In regard to fear, it emerged that it was the least recognized emotional category. Concerning the analyses carried out separately on each emotional category, the outcomes revealed that female participants better recognized the emotional categories of fear and happiness than male participants, thus underlining that there is an effect, albeit partial, of the participants’ sex in emotion recognition. Evidence suggests that behavioral response tendencies differ between men and women, potentially implicating evolutionary mechanisms. Investigating gender differences in affective research, and particularly in affective neuroscience, is crucial (Kret & De Gelder, 2012). As already stated, our study reveals that female participants demonstrate a greater accuracy in decoding emotions such as happiness and fear. This finding leads to the hypothesis that hormonal and neurological differences may be involved, as well as social learning, which may contribute to greater emotional awareness. It may also be related to cognitive differences found in the greater attention to visual details and better memory for facial expressions. However, these hypotheses require further investigation to achieve scientific validation. Regarding the participants’ age groups and therefore the observation relating to the possible differences in the three developmental stages examined, it emerged that adolescents decode disgust better than children, confirming this increase in recognition skills which becomes more refined towards adolescence, whereas pre-adolescents obtain worse performances in labeling neutrality than children and adolescents. In regard to the developmental stage of preadolescence, a brief digression is necessary to appropriately comment on the results obtained. In the literature, there are several studies focused on emotional recognition by children and adolescents. Unfortunately, the development of recognition skills by pre-adolescents has surprisingly not received the same attention and has been poorly investigated. A leading study in this field is the one conducted by Motta-Mena and Scherf (Motta-Mena & Scherf, 2017). The authors suggest that in the pubertal phase the perception of complex social expressions is modeled with particular care but that the same process does not occur for the perception intended for basic expressions. In regard to the emotional recognition abilities of pre-adolescents, it emerges that they are able to categorize happy expressions, followed by angry, disgusted, and neutral expressions and that they are not as skilled in recognizing sad and fearful expressions. Regarding the expression of neutrality, in the aforementioned study by Mancini and colleagues (Mancini et al., 2018), it is highlighted that pre-adolescents accurately recognize neutral expressions. Conversely, previous studies estimate neutral faces as emotionally ambiguous for samples consisting of both children and adults, often being mistakenly labeled as negative expressions (Lee et al., 2008; Waters et al., 2010). The results of the present study statistically demonstrate that pre-adolescents recognize neutral expressions significantly worse compared to children and adolescents, which is in line with the previous literature. Furthermore, observing the recognition percentage values reported in Table 1 and widely described in paragraph 3.3., although not significantly from a statistical point of view, they signal a weakening of recognition skills by pre-adolescents, especially for female participants, suggesting the strong need for further investigations. Another interesting aspect examined in this study concerns the effect of the stimuli’s sex. In a study performed by Esposito and colleagues (Esposito et al., 2018), aimed at exploring whether there were differences in emotion recognition between children and middle-aged adults, it emerged that with regard to the sex of the stimulus when emotions were represented by faces of male children they were better decoded, which is in contrast to studies in the literature that move in the opposite direction (Kret & De Gelder, 2012; McDuff et al., 2017). Although these results should be taken with caution, as stated by the authors, because it seems that the variable of the sex of the stimulus is strongly interrelated with other variables, such as emotional categories and participants’ sex. Our results, instead, are placed in an intermediate position, revealing that depending on the emotional category presented, stimuli conveyed by one sex are better encoded than the other. In fact, the analyses show that the facial expressions of disgust, sadness, and fear were better categorized when represented by female stimuli, while the facial expressions of surprise and neutrality were better categorized when portrayed by male stimuli. Concerning the stimuli’s ethnicity, our study does not seem to confirm the advantage of own race in the emotional recognition process, which is in line with (Reyes et al., 2018). To clarify, the Caucasian/European American ethnicity was found to be more poorly recognized for expressions of disgust and, respectively, better recognized for expressions of fear compared to all the other ethnicities. Concerning sadness, the Caucasian/European American ethnicity was better recognized than Latino and Asian ethnicities, with the exception of the African American ethnicity, which was best recognized than all in terms of sadness expressions. Moreover, the African American ethnicity was better decoded in terms of facial expressions of disgust than Latino and Asian ethnicities, whereas for facial expressions of surprise, the African American ethnicity was better recognized compared to Caucasian/European American and Latino ethnicities. Regarding the Latino ethnicity, the facial expressions of anger were more poorly decoded than those portrayed by Caucasian/European American and African American ethnicities, whereas the facial expressions of fear were more poorly decoded for African American and Asian ethnicities. For facial expressions of neutrality, the Latino ethnicity was better decoded than the African American ethnicity. Finally, the Asian ethnicity revealed to be the worst recognized in terms of facial expressions of anger among all ethnicities, and, moreover, the Asian ethnicity was more poorly recognized compared to Caucasian/European American and Latino ethnicities in terms of facial expressions of neutrality. From the emergent results, the influence of ethnicity on emotion recognition seems to depend on the emotional category presented, thus revealing rather heterogeneous patterns.

## 5. Conclusions

To conclude, the presented research could be helpful in different domains. Firstly, it contributes to a better understanding of emotional development. This study offers a comprehensive analysis of how emotion recognition skills evolve and become more refined during childhood, preadolescence, and adolescence. This knowledge is essential for understanding the stages of emotional and social development in young individuals. Secondly, the findings have educational implications. They can be used to design educational programs and interventions aimed at enhancing emotional skills in children, pre-adolescents, and adolescents. Furthermore, insights into sex and ethnic differences in emotion recognition can assist psychologists and therapists in tailoring their therapeutic approaches. For instance, specific interventions can be developed to support children struggling to recognize certain emotions. Additionally, the meta-analysis by Vannucci and colleagues highlights the link between intensive social media use among adolescents and risky behaviors, such as substance abuse and unsafe sexual practices. Peer influence and the pursuit of social acceptance exacerbate these dynamics, particularly on modern platforms like Instagram and Snapchat which emphasize social validation (Vannucci et al., 2020). Targeted interventions focusing on emotional education, emotional literacy, and self-regulation could play a crucial role in preventing critical situations and promoting the mental well-being of younger generations. Tackling these issues requires the combined efforts of families, communities, and institutions to create opportunities for positive development. By understanding how adolescents recognize and navigate their emotions, it becomes possible to identify early signs of distress and intervene to prevent adverse outcomes.

## Figures and Tables

**Table 1:** Percentage values of emotion recognition accuracy obtained from female and male children, pre-adolescents, and adolescents calculated on total recognition scores of each individual emotional category investigated. Please note: higher percentage values indicating better recognition accuracy are highlighted in bold, and they should be read in rows.

	Children		Pre-Adolescents		Adolescents	
	Females	Males	Females	Males	Females	Males
Disgust	**59.8%**	53.5%	12.5%	31.9%	**69.2%**	55.4%
Anger	**84.4%**	**76.5%**	11.5%	46.9%	**76.3%**	**68.8%**
Sadness	**75.4%**	**64.4%**	13.3%	39.4%	**64.4%**	55.8%
Fear	**47.7%**	36.3%	12.5%	24.8%	**48.3%**	36.5%
Happiness	**97.7%**	**89.4%**	12.5%	51.7%	**91.5%**	**75.8%**
Surprise	**90.8%**	**79.8%**	9.4%	48.1%	**86.9%**	**73.1%**
Neutrality	**74.8%**	**67.9%**	12.5%	27.5%	**67.5%**	**57.9%**

## Data Availability

The data presented in this study are available on request from the corresponding author. The data are not publicly available due to privacy reasons.
